# Beyond Joint Hypermobility: Investigating Bladder Dysfunction in Hypermobile Ehlers‐Danlos Syndrome

**DOI:** 10.1002/nau.70351

**Published:** 2026-06-18

**Authors:** Marium Ansari, Mallory Pine, Viktoriya Sapkalova, Emily Chan Brodowsky, Charles R. Powell, Ramzy T. Burns

**Affiliations:** ^1^ Indiana University School of Medicine Indianapolis Indiana USA; ^2^ Department of Urology Indiana University School of Medicine Indianapolis Indiana USA

**Keywords:** bladder dysfunction, connective tissue disorders, hypermobile ehlers danlos syndrome, lower urinary tract symptoms, pelvic floor dysfunction, urodynamics, voiding dysfunction

## Abstract

**Introduction and Objectives:**

Hypermobile Ehlers‐Danlos Syndrome (hEDS) is the most common subtype of Ehlers‐Danlos Syndrome, a group of connective tissue disorders caused by collagen abnormalities. While musculoskeletal features of hEDS are well characterized, its impact on visceral organs, including the bladder, remains underexplored. Despite frequent patient reports of urinary symptoms, a definitive link between hEDS and bladder pathology has not been established. This study aims to characterize lower urinary tract symptoms (LUTS) and urodynamic (UDS) findings in patients with hEDS to better understand potential mechanisms underlying bladder dysfunction in this population.

**Materials and Methods:**

A retrospective chart review was conducted on patients with hEDS who underwent video UDS at a single tertiary‐care center between 2022 and 2025. Inclusion criteria included age > 18, confirmed hEDS diagnosis (2017 criteria), and completed UDS. Data were analyzed using Welch's t‐test and Fisher's Exact Test.

**Results:**

Among 27 patients, the mean age at hEDS diagnosis was 30.9 years (range 18–49), 92.6% (*n* = 25) were female, and 92.6% (*n* = 25) were white. Common LUTS seen in these patients included frequency in 74.1% (*n* = 20), incontinence in 14.8% (*n* = 4), frequent urinary tract infections in 25.9% (*n* = 7), bladder pain in 33.3% (*n* = 9), nocturia in 48.1% (*n* = 13), and weak stream in 51.8% (*n* = 14) of patients. UDS findings revealed no evidence of detrusor overactivity, stress urinary incontinence, or abnormalities in compliance. 1 patient had incomplete emptying with a pre‐study post‐void residual of 500 mL. Pelvic floor dysfunction was seen in 18.5% (*n* = 5) with active EMG noted during emptying. Pelvic floor physical therapy was the most common intervention recommended, followed by beta‐3 agonists for overactive bladder symptoms.

**Conclusions:**

Our findings demonstrate that LUTS are prevalent in patients with hEDS, with evidence pointing to pelvic floor and neurologic dysfunction as key contributors rather than intrinsic bladder pathology. These results support prioritizing a pelvic floor‐focused approach to evaluation and management of hEDS‐related LUTS.

## 1 Introduction

Hypermobile Ehlers‐Danlos syndrome (hEDS) is the most prevalent subtype of the Ehlers‐Danlos syndromes (EDS), a heterogeneous group of heritable connective tissue disorders characterized by generalized joint hypermobility, chronic musculoskeletal pain, and multisystem involvement [1, 2]. In addition to musculoskeletal manifestations, individuals with hEDS frequently experience autonomic dysfunction, gastrointestinal dysmotility, pelvic organ prolapse, and neurological symptoms, including postural orthostatic tachycardia syndrome (POTS) [3, 4, 5, 6]. Spinal pathology is also frequently seen, including spinal instability, early disc degeneration and herniation, and tethered cord syndrome [3, 4, 5, 6]. Diagnosis of hEDS is clinical and based on the 2017 International Classification criteria, which require (1) generalized joint hypermobility, (2) specific musculoskeletal and systemic features, and (3) exclusion of alternative diagnoses [7].

Lower urinary tract symptoms (LUTS) are commonly reported by patients with hEDS; however, the underlying mechanisms remain poorly defined. Prior literature has largely focused on pelvic organ support disorders and patient‐reported urinary symptoms, with limited objective evaluation of bladder and voiding function [8, 9]. Proposed mechanisms include connective tissue abnormalities, autonomic dysfunction, and neuromuscular dysfunction of the pelvic floor [8, 10, 11, 12, 13, 14]. Type III collagen, which contributes to bladder compliance and viscoelastic properties, may be altered in hEDS, raising concern for impaired bladder storage or compliance [6]. However, objective evidence supporting intrinsic bladder pathology in this population is sparse.

Urodynamic studies (UDS) are the gold standard to evaluate lower urinary tract function, as allowing differentiation between storage and voiding dysfunction, evaluation of bladder compliance, and identification of detrusor overactivity or pelvic floor dyssynergia [8]. Despite frequent reports of LUTS in patients with hEDS, objective urodynamic data in this population are limited [10, 11]. As a result, urinary symptoms may be misattributed to nonspecific etiologies, potentially delaying targeted interventions.

The objective of this study was to characterize LUTS and urodynamic findings in patients with hEDS undergoing video urodynamic studies (VUDS) at a tertiary referral center. By defining objective patterns of bladder and pelvic floor function, we aim to improve the understanding of potential mechanisms underlying urinary dysfunction in hEDS and provide insight into clinical management strategies.

## 2 Materials and Methods

An institutional review board approval, a retrospective chart review was performed of adult patients with hEDS who underwent VUDS at a single tertiary care center between January 2022 and June 2025. All patients were referred for urodynamic evaluation by the same clinical geneticist. Inclusion criteria were age ≥ 18 years, a confirmed hEDS diagnosis based on the 2017 International Classification criteria, and completion of VUDS within the study period.

Demographic data were extracted from the electronic medical record. Clinical features associated with hEDS were collected based on documentation in the geneticist's consultation note and included joint hypermobility, muscle pain and weakness, postural orthostatic tachycardia syndrome (POTS), scoliosis, mast cell activation syndrome (MCAS), gastrointestinal symptoms, and other systemic manifestations.

Lower urinary tract symptoms prompting UDS were recorded, including urinary frequency, incontinence, recurrent urinary tract infections (UTIs), dysuria or bladder pain, nocturia, and weak stream or sensation of incomplete bladder emptying. Use of bladder‐directed medications at the time of UDS testing was also documented. Examination of pelvic support as determined by the POP‐Q system was recorded. Available spinal imaging was retrospectively reviewed.

All patients underwent standard VUDS performed in accordance with the International Continence Society recommendations [15]. UDS parameters collected included first sensation, first desire to void, strong desire to void, and maximum cystometric capacity (all measured in milliliters). Bladder compliance was calculated in cc/cmH_2_O. The presence of detrusor overactivity during the filling phase and urodynamic stress urinary incontinence was recorded. Voiding phase parameters included voided volume, maximum urinary flow rate (Qmax, mL/sec), and detrusor pressure at maximum flow rate (Pdet@Qmax, cm H_2_O). Electromyography (EMG) was used to assess pelvic floor muscle activity during filling and voiding phases. Video fluoroscopic images were reviewed by the interpreting urologist to assess for vesicoureteral reflux, abnormal bladder contour, or the presence of bladder diverticula.

Urodynamic interpretations, including presence or absence of detrusor overactivity, stress urinary incontinence, voiding dysfunction, or abnormal bladder compliance, were abstracted from the urologist's final report. Final clinical diagnoses (e.g., neurogenic bladder or pelvic floor dysfunction) and post‐study management recommendations were also recorded.

Descriptive statistics were performed using Microsoft Excel. Continuous variables are reported as means with standard deviations, and categorical variables are reported as frequencies and percentages.

## 3 Results

A total of 27 patients with hEDS underwent VUDS at a single tertiary care center between January 2022 and June 2025. The mean age at hEDS diagnosis was 30.9 years (range 18–49), and the majority of patients were female (92.6%) (Table 1). Comorbid conditions commonly associated with hEDS included POTS in 88.9% of patients, muscle weakness in 85.2%, and MCAS in 77.8% (Table 2). Additional comorbidities included endocrine/metabolic disorders (5, 18.5%), gastrointestinal disorders (3, 11.1%), autoimmune/inflammatory disorders (3, 11.1%), respiratory disorders (2, 7.4%), vascular abnormalities (2, 7.4%), neurologic/neurodevelopmental disorders (2, 7.4%), musculoskeletal abnormalities (2, 7.4%), and gynecologic disorders (2, 7.4%). Individual diagnoses included type 2 diabetes mellitus, obesity, thyroid disease, eosinophilic gastritis, chronic pancreatitis, ankylosing spondylitis, asthma, deep venous thrombosis, iliac vein compression syndrome, temporomandibular joint dysfunction, cervical instability, and polycystic ovarian syndrome.

**Table 1:** Patient demographics of hEDS patients.

Variable	All hEDS patients *n* = 27 (% or SD)
Sex	
Male	2 (7.4)
Female	25 (92.6)
Race	
White	25 (92.6)
Asian	1 (3.7)
More than one race	1 (3.7)
Ethnicity	
Hispanic or Latino	1 (3.7)
Not Hispanic or Latino	26 (96.3)
Mean BMI (SD)	27.43 (6.87)
Mean Age at time of hEDS diagnosis (SD)	30.93 (8.26)
Mean Age at time of UDS (SD)	33.04 (8.60)

Abbreviation: SD, standard deviation.

**Table 2:** hEDS characteristics of hEDS patients.

Variable	All hEDS patients *n* = 27 (%)
Joint Issues	
Hypermobility	27 (100)
Joint dislocation	15 (55.6)
Joint subluxation	17 (63.0)
Joint pain	26 (96.3)
Frequent sprains	11 (40.7)
Muscle pain	25 (92.6)
Muscle weakness	23 (85.2)
Postural orthostatic tachycardia syndrome (POTS)	24 (88.9)
Scoliosis	14 (51.9)
Mast cell activation syndrome (MCAS)	21 (77.8)
GI Issues	20 (74.1)
IBS	11 (40.7)
GERD	5 (18.5)
Gastroparesis	4 (14.8)
Chronic fatigue	23 (85.2)
Headaches/migraines	27 (100)
Neuropathy	13 (48.1)
Fibromyalgia	5 (18.5)
Anxiety	21 (77.8)
Depression	18 (66.7)
Eye problems (detached retina)	2 (7.4)

Among 19 patients with available spinal imaging, 70.3% demonstrated at least one abnormal finding. No patients had documented tethered cord syndrome or cervical instability. However, several patients demonstrated cervical and/or lumbar degenerative changes, including disc bulges, foraminal stenosis, retrolisthesis, spondylolisthesis, and nerve root compression. Additional findings included dural diverticula and a Chiari I malformation in one patient.

Patients reported a range of LUTS, with urinary frequency being the most frequently reported and the most prevalent (74.1%) (Table 3). VUDS demonstrated a mean first sensation volume of 122.26 ± 104.34 mL (Table 4). One patient exhibited incomplete bladder emptying with a pre‐study post‐void residual of 500 mL. The most frequent urodynamic abnormality was persistent pelvic floor electromyography (EMG) activity during voiding, observed in 66.7% of patients. Based on overall VUDS findings, 18.5% were diagnosed with pelvic floor dysfunction (PFD). No patients demonstrated detrusor overactivity, urodynamic stress urinary incontinence, vesicoureteral reflux, or abnormal bladder compliance (mean 77.33 ± 28.14 cc/cmH_2_0) on VUDS. Available POP‐Q examinations demonstrated Stage II prolapse in only 2 patients. Consistent with urodynamic findings, the most common post‐study management recommendations were pelvic floor physical therapy (40.9%) and beta‐3 adrenergic agonists (22.2%).

**Table 3:** Baseline bladder characteristics of hEDS patients.

Variable	All hEDS patients *n* = 27 (% or SD)
Urinary frequency	20 (74.1)
Mean of frequency during daytime (every X hours)	2.07 (1.29)
Urinary Incontinence	4 (14.8)
Stress	2 (7.4)
Urge	2 (7.4)
Frequent urinary tract infections (UTIs)	7 (25.9)
Dysuria or Bladder Pain	9 (33.3)
Nocturia	13 (48.1)
Mean how often at night (X times)	3.08 (2.15)
Weak Stream/Sensation of Incomplete Bladder Emptying	14 (51.9)

Abbreviation: SD, standard deviation.

**Table 4:** Urodynamic parameters for hEDS patients.

Baseline bladder characteristics	All hEDS patients *n* = 27 (% or SD)
First sensation (mL)	25.74 (21.06)
First desire (mL)	122.26 (104.34)
Strong desire (mL)	251.84 (197.92)
Maximum cystometric capacity (mL)	422.56 (204.39)
Compliance (cc/cmH20)	77.33 (28.14)
Stress Incontinence	0
Voided volume (mL)	320.42 (212.78)
Qmax (mL/second)	14.13 (16.69)
Pdet (cm H20)	12.27 (13.89)
EMG while voiding	
Active	18 (66.7)
Relaxed	6 (22.2)
Unknown	3 (11.1)
Detrusor Overactivity	0
PVR (mL)	95.89 (203.82)

Abbreviations: Pdet, detrusor pressure at Qmax; PVR, post‐void residual volume; Qmax, maximum flow rate; SD, standard deviation.

## 4 Discussion

In this cohort of patients with hEDS, LUTS were common, with urinary frequency reported most frequently. Despite this symptom burden, VUDS demonstrated preserved bladder storage function, with normal bladder compliance and no evidence of detrusor overactivity or vesicoureteral reflux. In contrast, the most consistent VUDS abnormality was persistent pelvic floor EMG activity during voiding, observed in a majority of patients. This finding suggests that pelvic floor neuromuscular discoordination, rather than intrinsic bladder pathology, may play a central role in LUTS among patients with hEDS. The high prevalence of abnormal EMG activity indicates that either subclinical or functional pelvic floor dysfunction may be common in this population. These findings align with prior descriptions of connective tissue laxity and neuromuscular incoordination in hEDS and warrant careful clinical consideration [3, 4, 5, 8, 11].

A notable finding was the discordance between symptom burden and objective bladder storage abnormalities. Despite frequent urinary symptoms, patients with hEDS demonstrated preserved bladder storage parameters and lacked the classic urodynamic findings commonly seen in idiopathic overactive bladder or neurogenic bladder populations, such as detrusor overactivity, impaired compliance, or vesicoureteral reflux. This pattern suggests that LUTS in hEDS may reflect a distinct clinical phenotype characterized by preserved bladder storage parameters despite significant urinary symptom burden. Potential mechanisms include pelvic floor neuromuscular discoordination, altered visceral sensory processing, autonomic dysfunction, and connective tissue‐related alterations in pelvic floor support.

The high prevalence of associated comorbidities, including POTS and neuropathic symptoms, further supports a multifactorial pathophysiology distinct from idiopathic lower urinary tract dysfunction. In addition, spinal pathology has been increasingly recognized in hEDS. Although no patients in our cohort had documented tethered cord syndrome, several demonstrated cervical or lumbar degenerative changes, foraminal stenosis, and disc protrusions on available imaging. While the clinical significance of these findings remains uncertain, they further support the possibility of neurologic contributors to LUTS in this population.

Although type III collagen contributes to bladder wall structure and compliance, impaired compliance was not observed in our cohort. Likewise, vesicoureteral reflux and structural abnormalities were rare. The findings suggest that collagen‐related bladder wall abnormalities in hEDS, if present, may be subclinical or not readily detected by conventional urodynamic measures. Instead, LUTS in hEDS may be driven by functional, neuromuscular, or sensory mechanisms rather than overt structural bladder pathology. These findings may help explain why many patients with hEDS continue to experience LUTS despite the absence of classic urodynamic abnormalities and suggest that negative urodynamic findings should not preclude further evaluation of pelvic floor and neurologic contributors.

These findings suggest that routine UDS may not consistently demonstrate abnormalities in hEDS patients, even in the setting of significant LUTS. Clinicians should therefore exercise caution in attributing symptoms solely to intrinsic bladder pathology alone. Although only two patients demonstrated Stage II prolapse on available POP‐Q examinations, persistent pelvic floor EMG activity was common, suggesting that pelvic floor dysfunction in hEDS may be primarily neuromuscular rather than structural in nature. These findings support early referral to pelvic floor physical therapy and a multidisciplinary treatment approach.

While this study is among the first to systematically evaluate LUTS and UDS findings in patients with hEDS, several limitations should be acknowledged. The retrospective design, small sample size, and single‐center nature of the study limit generalizability. The absence of a matched control population limits direct comparison between hEDS‐associated LUTS and idiopathic lower urinary tract dysfunction. All patients were referred by a single clinical geneticist, introducing the potential for referral and selection bias. In addition, although UDS are considered the gold standard for evaluating lower urinary tract function, they represent a snapshot in time and may not capture subtle, intermittent, or context‐dependent dysfunction. Finally, the absence of validated symptom questionnaires and a control population limits correlation between subjective symptom burden and objective urodynamic findings.

## 5 Conclusion

Patients with hEDS may represent a distinct LUTS phenotype characterized by significant urinary symptom burden despite largely preserved bladder storage function on urodynamic testing. Our findings suggest that pelvic floor neuromuscular dysfunction and neurologic contributors, rather than intrinsic bladder pathology, are primary drivers of urinary symptoms in this population. These results support a pelvic floor‐focused and multidisciplinary approach to the evaluation and management of LUTS in patients with hEDS.

## Funding

The authors have nothing to report.

## Ethics Statement

This retrospective chart review was approved by the Indiana University Institutional Review Board (Approval No. 27453). The study was conducted in accordance with institutional ethical standards and the principles of the Declaration of Helsinki. Given the retrospective design and use of de‐identified data, the requirement for informed consent was waived by the Institutional Review Board. This study was a retrospective chart review and did not involve prospective assignment of interventions; therefore, clinical trial registration was not required.

## Conflicts of Interest

The authors declare no conflicts of interest.

## Permission to Reproduce Material

No previously published figures, tables, or copyrighted materials were reproduced in this manuscript.

## Data Availability

The data that support the findings of this study are available from the corresponding author upon reasonable request. The data that support the findings of this study are not publicly available due to patient privacy and institutional ethical restrictions. De‐identified data may be made available from the corresponding author upon reasonable request and with appropriate institutional approval.
